# Behind the screen: exploring the effects of home working on 999 telephone clinicians during the COVID-19 pandemic

**DOI:** 10.29045/14784726.2024.9.9.2.1

**Published:** 2024-09-01

**Authors:** Edward Harry, Mike Brady

**Affiliations:** Welsh Ambulance Services University NHS Trust ORCID iD: https://orcid.org/0000-0003-4092-6134; Welsh Ambulance Services University NHS Trust ORCID iD: https://orcid.org/0000-0001-6675-9149

**Keywords:** 999, ambulance, health, home working, pandemic, remote, telephone consultation, well-being

## Abstract

**Introduction:**

The COVID-19 pandemic has significantly stretched global healthcare provisions since its commencement in 2019. From the outset, ambulance services in the UK had to adapt and change their working practices to meet distancing requirements, to increase staff numbers and to ease the effects of staff becoming unavailable for work due to self-isolation and illness. One strategy was moving clinicians from emergency operation centres (EOCs) to working from home. Like many international services, UK ambulance services use paramedics and nurses to undertake telephone and video assessments of patients calling the 999 emergency services line in a model known as virtual care or remote clinical decision making. Virtual care is any interaction between a patient and a clinician or clinicians, occurring remotely via information technologies.

Increasing evidence is becoming available to suggest that the pandemic caused harm to the well-being of healthcare workers, primarily due to the severe stress of regular exposure to death and human suffering. However, there remains a dearth of literature focusing on the well-being of remote and virtual clinicians, especially those who moved from working in EOCs to working at home during the COVID-19 pandemic. Therefore, this study reports the findings of a qualitative analysis of these effects from the clinician’s perspective. The authors hope that the findings from this study will inform the operating, well-being and leadership practices of those delivering such services.

**Methods:**

A convenience sample of telephone nurses and paramedics from one UK ambulance service where home working had been implemented were contacted. Fifteen clinicians with recent home-working experience responded to the invitation to participate out of a possible 31 (48%). All participants had previously practised remote assessment from within an EOC. Semi-structured interviews took place via video-conferencing software and were recorded, transcribed and thematically analysed. An inductive approach was taken to generating codes, and both researchers separately read the transcripts before re-reading them, assigning initial themes and determining frequency.

**Results:**

Five main themes were discovered, with further associated sub-themes. The main themes were: safety; financial implications; working relationships; home-working environment; and anxiety.

**Conclusions:**

Few studies explore remote clinicians’ health and well-being. This study identified that home-working clinicians felt that there had been no detrimental impact on their health and well-being because of working from home during the initial phase of the COVID-19 pandemic. While some concerns were raised, these were mitigated through the support that clinicians received at home from family members, as well as from colleagues, some of whom had developed new working relationships. Financial implications appeared to have contributed to some concerns for participants initially, but these had been alleviated quickly despite requiring further exploration of the true financial impact of working from home.

## Introduction

The COVID-19 pandemic has significantly stretched global healthcare provisions since its commencement in 2019. At the forefront of the changes that the pandemic brought about were ambulance services across the UK, which played a vital role in the healthcare system by providing support in situations that directly threatened patient health and life (Piotrowski et al., 2021). More evidence has become available recently to suggest that the pandemic has had a detrimental effect on the mental health and well-being of healthcare workers, primarily due to the severe stress of the regular exposure to death and human suffering that was more prevalent at the time (Marczewski et al., 2021). These healthcare workers include those involved in virtual care or remote clinical decision making (RCDM) (Brady & Harry, 2023). Virtual care is any interaction between a patient and a clinician or clinicians occurring remotely via information technologies (New South Wales Government, 2021).

RCDM staff comprise a range of healthcare professionals, including paramedics, nurses and pharmacists, all of whom provide remote secondary triage to patients calling the 999 emergency service line (Brady & Harry, 2023). The COVID-19 pandemic saw 999 emergency services adapting and changing their working practices to meet distancing requirements, increase staff numbers and ease the effects of staff becoming unavailable for work due to self-isolation and illness (Tang et al., 2020). One such strategy involved moving clinicians from emergency operation centres (EOCs) to working from home, utilising remote computer-aided dispatch modules, remote clinical-decision support software, digital phone systems and video-based platforms, which allowed close to full functionality compared with typical EOCs (Tang et al., 2020).

Currently, while the research surrounding front-line healthcare workers and the impact that the pandemic had on them grows, there is a paucity of evidence exploring the health and well-being of RCDM workers, especially in the context of UK 999 ambulance services, specifically concerning staff who moved from EOCs to home-working models. Therefore, this study reports on the qualitative analysis of the effects on the health and well-being of RCDM workers from one UK ambulance service while they were working from home during the initial phase of the COVID-19 pandemic. It is hoped that the findings from this study will inform operational, health and well-being, and leadership strategies across various services, especially for organisations that continue to operate with pandemic-like models, such as home working, in place in some form.

## Methods

A convenience sample of RCDM (telephone) nurses and paramedics from one UK ambulance service where home working had been implemented were invited to participate in a semi-structured interview. Participants were selected if they were registered nurses or paramedics, had been working from home for more than one week during the pandemic and had previous experience working in an EOC. No further experience or qualification criteria were applied. No participants had been identified as being re-deployed from other duties within the service. Email adverts and information sheets were sent to a group of prospective participants, asking those interested to contact the researcher. The participating ambulance service covers a 20,000-kilometre area and serves a population of nearly three million people: rural, coastal, large city and urban districts. Given the generalisability of the sample ambulance service area and that, at the time, many services were still reticent to be involved in any work that was not directly associated with the pandemic response, only one service was approached. Fifteen clinicians (working a range of shift times and patterns) with recent home-working experience responded to the invite out of a possible 31 (48%). All participants had previously practised remote assessment from within an EOC, using only telephone consultation at the time, as video was introduced later. Informed consent was gained using the information sheet and signed consent forms.

This article forms the second part of a two-part series exploring the impacts on well-being and telephone consultation practice (Brady & Harry, 2023). Approval was sought via the Integrated Research Application System (IRAS number: 287,662) and approved by the National Health Service Health Research Authority. Ethical approval was not required for this study, as the participants involved staff members.

### Data collection and analysis

**Figure 1:**
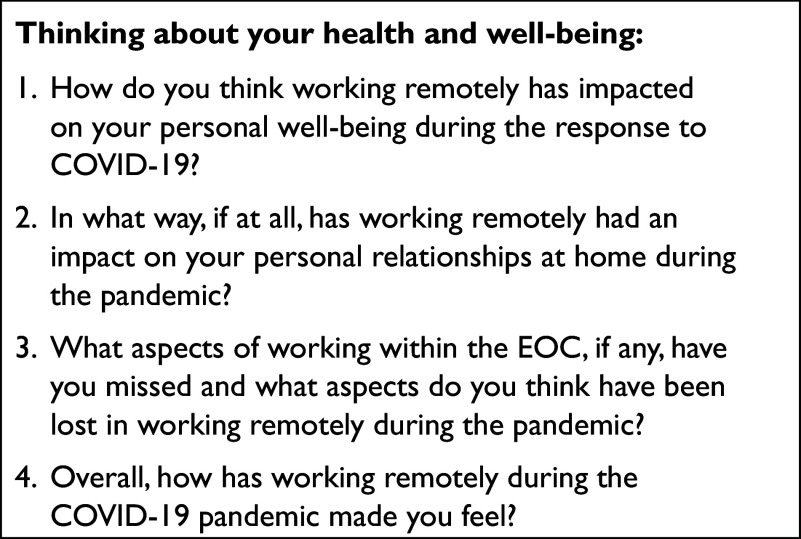
Figure 1. Semi-structured interview guide.

Due to COVID-19 safety restrictions, semi-structured interviews were conducted by a lone researcher utilising a semi-structured interview guide (Figure 1) via remote video-conferencing software. Interviews were recorded, professionally transcribed and thematically analysed into codes using Braun and Clarke’s (2006) frameworks for thematic analysis. No computer-assisted software, such as NVivo, was utilised.

The term ‘coding’ refers to an idea or issue relevant to analysing the data and can relate to a number, phrase or word (Denscombe, 2021); coding was undertaken to capture the raw data relevant to the topic of enquiry. Data saturation was not sought by the researchers, owing to the size of the convenience sample. Furthermore, due to COVID-19 still being in its infancy at the commencement of the study, it was considered pertinent to gain as much information on the effects of COVID-19 on participants as possible.

The researchers used an inductive approach to generating codes to reduce research bias. Both researchers read the transcripts separately and re-read them prior to identifying initial themes and determining frequency. The researchers then met to discuss the themes identified and to compare and contrast any nuances. This approach ensured that all interview areas were reviewed, and all themes explored and identified were discussed and included or excluded. It also ensured that any themes not identified by the other researcher could be introduced, discussed or challenged.

Further discussions generated code labels and sub-theme identification for those codes, which were varied and nuanced. Following this, the researchers discussed and revised the codes through several iterations. This process took place over approximately eight weeks, ensuring that the researchers had the time and capacity to reflect on the transcripts and subsequent themes and codes.

## Results

Data saturation was not met due to the low sample size, as only 15 participants came forward to take part, despite reminder emails and further adverts.

Table 1 outlines the participants’ professional registration and the number of years they had been qualified. No individual was qualified for fewer than six years or more than 40 years.

**Table 1.** RCDM demographics.

Profession	Total	Years qualified	
**Nurse**	8	6–10 years	3
		11–20 years	1
		21–30 years	2
		31–40 years	2
**Paramedic**	7	6–10 years	3
		11–20 years	2
		21–30 years	1
		31–40 years	1

Five main themes were identified with their associated sub-themes. These themes were:

1. Safety
2. Financial implications
3. Working relationships
4. Home-working environment
5. Anxiety.

### Theme 1: safety

Safety was described as participants feeling that they and those close to them were at reduced risk or kept free from COVID-19 infection or ill health. They noted that safety was a serious concern of theirs and had not been a concern prior to the pandemic. It was remarked that they found an increased likelihood of catching COVID-19 if they worked within the EOC. Working from home, participants felt safer and could create a unique environment, different from that in the EOC.

*I feel much more . . . much safer to be at home.* (Nurse 3, 40 years qualified)

*I personally feel safer at home because that’s my own little bubble.* (Paramedic 9, six years qualified)

Participants spoke about their family in detail. Some participants noted that they felt at ease working from home due to being able to protect their clinically vulnerable family members.

During the COVID-19 pandemic restrictions, government advice included recommending those with clinically vulnerable conditions such as asthma, those undergoing active cancer treatments and those with immunosuppressive disorders to shield themselves from risk. It was evident that RCDM clinicians did not want to increase the likelihood of contracting COVID-19 and bringing it home to their families.

*Because I don’t really want to expose myself to more people than necessary, because I look after my mum who is clinically vulnerable.* (Paramedic 11, 12 years qualified)

*My husband has multiple problems, so the worry of me going to work and bringing something home to him was quite overpowering, so yes it’s made a massive difference for me to be able to keep us both safe at home.* (Nurse 3, 40 years qualified)

*You know, I’ve got elderly parents, you know, I’m the one who goes over there. So the risk there is pretty high.* (Nurse 7, 10 years qualified)

### Theme 2: financial implications

Participants noted the impact that working from home placed on them financially. While it was mentioned that there was a reduction in some fuel costs, home energy costs had increased due to home working, but at the time of interviews, many had not yet seen a difference in expenditure.

*You don’t have to get up so early, you’re saving money on fuel. Although heating costs, especially during the winter, could have increased. So it’s swings and roundabouts from a financial perspective.* (Nurse 7, 10 years qualified)

Similarly, staff members noticed that the reduction in travelling time had an impact. Staff members prior to COVID-19 would be required to travel to the EOC for the start of their shift and travel home upon completion of the shift. It was noted that this travelling time would impact on fuel costs.

*And again, you know, for me, living in [. . .], having to travel to [. . .], it’s about 40 minutes each way. I suppose you’re finishing your shift and you’re home, you’re already home. So it’s less fuel money.* (Paramedic 5, 27 years qualified)

### Theme 3: working relationships

Participants often spoke about their previous working relationships while working within the EOC, compared to their new working relationships when working from home, which at times involved new methods of communication, including increased use of remote video software.

Participants also noted that they were more likely to contact other staff members to check on their well-being, perhaps more so than when in an EOC together. It was also suggested that the pandemic had brought colleagues closer together, which was positive for well-being.

*I don’t know, if you have this sense of duty to make sure that everyone is okay, and perhaps the actual pandemic has brought us closer as a team because we know how much pressure each and every one is under.* (Nurse 1, 10 years qualified)

Participants also observed that as a result of home working, they had developed professional working relationships with members of staff with whom they may not have interacted prior to the COVID-19 pandemic.

*I’ve probably never communicated with other sites unless I needed, whereas now because we’re all working remotely, it doesn’t seem to matter where anyone is. So I’ve got to know staff from other EOCs much more than I probably would have.* (Nurse 15, 15 years qualified)

Participants also noted an absence of working relationships by not being in the EOC, which was a less positive experience.

*So I think that kind of interaction between people, there’s a lot of interaction normally when you’re working in EOC at the clinical desk with the allocators, the call takers if that’s, you know, or giving advice as a clinician to non-clinical staff within the EOC sector. All of them aspects are lost.* (Paramedic 14, 35 years qualified)

Before the pandemic, staff would work alongside each other in an EOC, bringing a sense of professional relationship. Some participants felt this was lost from the RCDM clinicians and other staff members working within the EOC, such as emergency medical dispatchers (EMDs).

*I don’t know whether any of our colleagues from allocated dispatch would say that they’ve missed having our contact. But I think, you know, we all – we’re all here to work as a team, and that’s one thing that I’ve noticed.* (Paramedic 2, 10 years qualified)

Participants did note, however, that they felt that online platform meetings soon ran their course and that ‘Zoom fatigue’, as it was coined, set in. Conversely, participants also highlighted that online social meetings enabled them to develop working relationships with individuals they would not have routinely met due to geographical constraints.

*Going on Zoom or Teams was great to start with, technology is great, but I did get fed up of it really quick. I get it helped with work and meet [sic] people that were the other side of the country, but I did get fed up of it by the end.* (Paramedic 11, 12 years qualified)

### Theme 4: home-working environment

The home-working environment was discussed in the contexts of creating a dedicated space for work in the home, the challenge of feeling as if one is living in an office environment and the need to create a sense of separation between work and home.

As many people experienced across the UK during the initial stages of the stay-at-home instructions issued by the government, a period of adaptation was required for individuals to transform their home environment into a working environment. This appeared to be easy for some participants and less so for others. A sense of separation between work and home life was often important for their well-being.

*When I finish my shift, I have to literally shut everything down, put it all way. I always go out for a walk and then when I come back, my kitchen’s my kitchen again.* (Nurse 3, 40 years qualified)

*I made a conscious effort to separate my home and workplace.* (Nurse 4, 32 years qualified)

This ability to practically separate working life and home life appears to have supported participants’ ability to unwind after shifts. Working from home, clinicians felt their relationships with their families had strengthened. Moreover, stay-at-home guidance from the government meant that families had the time and contact to spend with one another.

*I probably think that we’ve got closer. Like I say, my daughter’s 19. So in normal times she’d be out and about, but, you know, she’s at home more. I see my husband more. So, and I think it’s brought us closer.* (Nurse 4, 32 years qualified)

Furthermore, domestic relationships were seen to have improved, with some clinicians noting that they were not arguing as much due to the agile working.

*And believe it or not, we’ve both of us have been working from home for the majority of the time, I can’t get over how little arguments there’s been in the house.* (Paramedic 14, 35 years qualified)

Participants also felt that they could support their families in home-working life.

*And I suppose it grants you the opportunity if you’re in your breaks and your screen-breaks, to help out. We’ve got a young two-and-a-half year old, who’s fairly intense. So you know when I’m taking a five-minute screen-break, or my half an hour’s lunch, I can help out, I can give my wife a break.* (Paramedic 2, 10 years qualified)

### Theme 5: anxiety

Participants described their anxiety about returning to EOC-based environments and the perceived risk this carried. They also expressed their feelings of anxiety at never being able to return. The concept of gradual adjustment was noted throughout.

*The thought of going into the contact centre worries me. I feel anxious. While this pandemic is around, I would be very reluctant to go back because of being in an enclosed environment with people with potential COVID.* (Paramedic 11, 12 years qualified)

Conversely, some individuals felt that it was time to return to the normal working environment of the EOC.

*I think there’s got to be a term of adjustment now, and as things change and move forward in inviting clinicians into the call centres, there’s got to be an adjustment.* (Nurse 3, 40 years qualified)

## Discussion

### Safety

The feeling of safety was important to participants and created a sense of well-being for them and their families. Participants discussed how there had never been a concern regarding safety within the EOC before the pandemic. While many noted that working from home increased the perception of safety in a ubiquitously unsafe pandemic environment, this would be difficult to quantifiably measure in the absence of pre-home-working assessments. A study conducted by Chen et al. (2023) that assessed home working prior to the pandemic did not mention safety as an important factor in achieving work outcomes, and no study has been identified that raises safety as a concern for those working from home. Thus, it might be argued that home working has not been considered in terms of mental well-being before. Similarities to the environment created by COVID-19 safety, however, could be applied to those who are immune suppressed and are more susceptible to disease, noting that the extent to which participants adhered fully to the government rules at the time, when not working, were not measured. Participants were health workers, and it might be argued that their exposure to information, policies, clinical guidelines and the response to COVID-19 itself led to a greater sense of insecurity to begin with (Ammar et al., 2020; Musgrove et al., 2022), especially when seeing many hundreds of cases on remote computer-aided dispatch screens.

Brehon et al.’s (2022) cohort study concluded that, while there was no guaranteed way to ensure the safety and well-being of home workers, there appeared to be a general opinion that individuals staying safe and minimising contact with others would be beneficial. The safety of others, including the home workers’ loved ones, which, inevitably, would lead to improved well-being, was also highlighted and, in turn, it was emphasised that this reduced feelings of social isolation (Brehon et al., 2022). For example, the concept of safety from infection and illness was not solely restricted to COVID-19, and participants reflected on their previous approaches to illnesses, such as seasonal flu. Indeed, Dhanasekaran et al. (2022) discussed the way in which global COVID-19 restrictions had heavily suppressed flu circulation, and that, while this was positive for individuals, some consequences, such as social isolation and unfamiliarity, needed to be considered more broadly. These findings suggest that home-working models might be adapted to support health-related, seasonal and future outbreak situations in immune-suppressed staff during flu season or local outbreaks of viral infections, such as norovirus, which could arguably lead to increased well-being (Okuyan & Begen, 2021).

### Financial implications 

Participants highlighted the financial impact of working from home, emphasising a saving in car fuel and a reduction in commuting time, while also considering the rise in their usage of domestic gas and electricity. Indeed, some individuals commented that they no longer had to commute long distances to the EOC, which reduced their financial expenses for car fuel and provided them with better financial security during an unpredictable time. Links between financial security and well-being have close ties with one another (Chu et al., 2022), so it is unsurprising to find that participants presented financial considerations as discussion points during the interviews and explored the close links between finances and the improvement of well-being.

There was little literature exploring the reduction in costs for home-working staff and the resultant impact of this on well-being. However, Giovanis & Ozdamar (2022) concluded in their study analysing the empirical implications on households that, while the financial situation for home workers during the COVID-19 pandemic was better compared to those who had never worked from home, there appeared to be no difference in their mental well-being. During this study, however, participants did also note that it was too early to determine the true financial impact of working from home, although they overwhelmingly felt that they had seen a reduction in their financial costs, which positively impacted their well-being. It should also be considered that individuals did not have other financial expenditures associated with leaving their home, such as visiting restaurants or other outlets that were closed due to lockdown measures being in place, so perhaps individuals felt more financially secure in general.

The UK has recently seen many industries affected by strike action (White, 2022), as staff attempt to gain better pay and working conditions. Many of the same industries are attempting to recover the cost of the COVID-19 response and are trying to shelter themselves from the effects of the volatile financial outlook (White, 2022). If home working continues to positively impact financial security and well-being in a group of highly skilled and highly sought-after clinicians, then organisations, managers and employees might view it as a positive step towards improved working conditions and higher disposable income. Therefore, more up-to-date research in this area may be required, particularly as the incumbent cost-of-living crisis has been shown to have such a detrimental impact on the well-being of society (Barnett, 2023).

### Working relationships

Social interaction creates a positive stimulus for an individual (Coşkun et al., 2022) and can improve team well-being. However, during the pandemic, this was disrupted. Working relationships are key relational mechanisms that have been noted to build trust and can transcend into the manner in which an individual operates within their daily tasks (Toniolo-Barrios & Pitt, 2021). Participants noted the physical absence of the colleagues who normally supported them in the EOC environment. Individuals discussed that the physical presence of others often supported them, both in their capacity to undertake their duties and on a personal level. This finding is also supported by Brady and Harry’s (2023) qualitative study of RCDM workers and the effects that home working had on telephone triage practice, being one that might lead to poorer well-being.

Workplace isolation while working from home is thought to impact well-being if experienced negatively, and it is considered a significant barrier to implementing fully remote models (Galanti et al., 2021). This cross-sectional study, however, identified that many participants did not feel isolated and, in fact, felt that their families provided comfort and made them feel safer. Moreover, the physical absence of colleagues was replaced with individuals needing to support one another while undertaking home working in different, more virtual ways, which appeared to positively impact their health and well-being. This included regular telephone conversations and newly generated weekly coffee morning meetings on online digital platforms, where individuals discussed matters unrelated to COVID-19 or work.

Peer support is a common practice within ambulance services. Carvello et al. (2019) identified peer support as a profound coping mechanism during emotional labour and in stressful working environments. A study by Musgrove et al. (2022), involving an online survey of healthcare professionals, observed that peer support was found to have supported participants, particularly in feeling less isolated and alone and in having a safe space to discuss issues. It became apparent, however, that such techniques were time limited. These findings suggest that a balanced approach is required from organisations and managers, and that a blended or mixed approach needs to be taken to offer peer and team support. This position is supported somewhat by the Sommerlad et al. (2021) questionnaire study, which noted that frequent face-to-face contact and phone/video contact were linked to lower levels of depression and anxiety during the initial stage of the pandemic (March–August 2020).

This study focused solely on the participants and how home working affected their health and well-being. However, they were willing to openly discuss their concerns over the health and well-being of the individuals they usually worked with in the EOC. These included non-clinical staff operating within the EOC and ambulance call takers, dispatchers and EOC managers. Due to the often unpredictable and stressful environment in which ambulance service workers operate, individuals often create close relationships with those not of the same grade (Miller, 2021). It is believed that the pandemic has encouraged this ethos (Association of Ambulance Chief Executives (AACE), 2021). Participants highlighted that non-clinical staff working to provide a service to patients could be heavily reliant on RCDM in the EOC on occasion. This study is limited because it did not explore the views of other staff roles in EOCs.

### Home-working environment

Coşkun et al.’s (2022) cross-sectional analysis found that an estimated four in ten European workers started working from home during the pandemic. This shift in working conditions allowed many operations models to continue, reduced stress in the working environment and increased family time. It made it easier for workers to maintain better and healthier lifestyles. That being said, Ammar et al. (2020) previously highlighted that prolonged time spent in this workspace environment could lead to other mental conditions, such as social isolation, and considered whether individuals were at risk of developing return-to-work anxiety or agoraphobia. These matters were discussed in separate themes.

Participants highlighted that they had developed and set up a working environment tailored to their needs, including office spaces within their kitchens or garages. Ammar et al.’s (2020) review of the literature found that home workspace confinement could negatively affect mental well-being, mood and feelings during COVID-19, and it raised concerns over whether an individual can truly separate their workspace from their home space. Participants found ways to adapt to this concern, however. They discussed how they could separate their workspace from their home space very well, often generating a routine or pattern that ensured they could distinguish between the two. Participants shared the ways in which they created a positive atmosphere, using a common phrase, ‘my bubble’. Interesting to note was the way in which participants felt that when numerous items, such as laptops and notepads, were placed away from sight, they felt instant relief. They felt that this assembly routine was associated with their productivity, a theme noted by Brady and Harry’s (2023) qualitative study into RCDM practice. The concept was further highlighted by Galanti et al. (2021) in their cross-sectional study, which found that when people felt more productive, they had improved well-being, reduced stress and were distracted from COVID-19. Some end-of-shift stressors were noted, but these reports appeared to be quickly dissipated by participants engaging in outdoor exercise or walking activities. Exercise, while limited to the outdoor setting, was observed to be a significant distraction for individuals during the COVID-19 pandemic and was actively encouraged, being one of the few reasons why they were permitted to go outdoors (AACE, 2021; Toniolo-Barrios & Pitt, 2021). These findings suggest that organisations running home-working models should explore how ‘pop-up’ workstations can be supported or how to include separation techniques in training and induction sessions.

Galanti et al.’s (2021) study noted that home workers were faced with the distractions and disruptions of a normal working household – primarily due to COVID-19 restrictions and guidelines. Participants noted that this was likely due to childcare and reorganising shift work around spouses who were required to continue working outside the home. Participants did not appear to think that this negatively impacted their well-being, but rather that their ability to be at home with their loved ones had a more positive influence. Participants felt this was an overwhelmingly positive contributor to improving or sustaining an optimistic outlook towards their health and mental well-being. As previously noted, their families supported them while working from home. Shin et al. (2021) noted a similar finding in their retrospective analysis study, where family support was found to have had a positive longitudinal effect on work outcomes for employees and helped in the avoidance of exhaustion and burnout. This ‘family factor’, as described by Brehon et al. (2022) in their descriptive cohort study, has been alluded to as having had both a positive and negative influence on home workers, and the authors questioned to what extent this may now have changed in a post-pandemic environment, especially as vaccinations became available. The possible sense of COVID-19 risk has decreased.

### Anxiety

The COVID-19 pandemic created a sense of the ‘unknown’ that was incomparable with any other modern example (Diab-Bahman & Al-Enzi, 2020). Little research or information was available to educate or appease individuals fearful of COVID-19 at the onset of the pandemic and the overwhelming presence in the media at the time was thought to be closely linked with individuals’ anxiety (Toniolo-Barrios & Pitt, 2021). Coşkun et al. (2022) identified that COVID-19 and working from home caused anxiety-related emotional consequences, including stress, depression, irritability, insomnia, fear, confusion, anger, frustration, boredom and stigma. This study, however, found differently. Very few participants experienced a sense of anxiety while working from home, which may suggest that more research is required to explore differences in variables such as occupation, age, sex and family constructs. It may also be important to consider whether individuals working within high-stress environments and roles in the ambulance service and then required to work from home during the pandemic had similar experiences to the RCDM or whether they experienced different stressors or emotional consequences that had been highlighted by Coşkun et al. (2022).

Platts et al.’s (2022) study noted that those who had experienced anxiety and anxiety-related symptoms when working from home often had a pre-existing mental health condition diagnosed or experienced personal trauma during the pandemic, such as losing a loved one, precipitating further anxiety. While this study only focused on a snapshot of RCDM staff working from home, it is unknown which participants, if any, experienced previous mental health conditions. This concept is important to consider, given that a previous survey conducted by MIND (2019) found 75.8% of ambulance workers to have personal experiences with mental health problems.

While participants in this study were anxious over their loved ones’ health, the predominant theme related to anxiety over returning to the EOC and the potential for increased risk of contracting COVID-19. Participants perceived that due to increasing operational demand and the complexity in responding to this demand, there were attempts at encouraging more blended approaches, which invariably meant returning to the EOC (providing they were not deemed at risk), which caused anxiety. Many organisations, including ambulance services, had adapted their workspace environments to make environments ‘COVID-safe’, with increased availability of personal protective equipment (PPE), such as Perspex screens and masks. Central government and devolved nations also supported these industries in providing them with guidance and checklists to reduce the spread of the virus (Urch & George, 2020).

Anxiety within healthcare working environments is not unusual, owing to the fast-paced and often unpredictable setting in which individuals operate (Carvello et al., 2019). Some participants noted that they tried to focus on a positive mindset rather than allowing detrimental thoughts to overcome them. This concept has been identified as being pre-emptive in ensuring individuals maintain a positive outlook and is not a new concept but one which might be applied to post-pandemic home-working models. Chu et al. (2022) concluded that if employees hold the mindset that stress and anxiety are debilitating, they will tend to focus on negative information from stressors, reinforcing pessimistic beliefs and causing them to take action to avoid the stressors. This would have been particularly relevant for home workers during this period as they were faced with the implications of the pandemic, both in their personal lives through social restrictions and in their working environments.

### Implications for practice

This article outlines the effects of home working on the well-being of EOC staff during the COVID-19 pandemic that organisations, managers and employees should note. It highlights the need to research the well-being of those who are no longer working in the same environment as them (EMDs). It demonstrates, in various areas, where pandemic practice requires adaptation for post-pandemic practice and where organisations, managers and employees can work to produce environments that aid the maintenance of positive well-being, both for individuals and for teams. Despite limitations, this study found no overtly concerning reason to pause or stop home working from a well-being perspective. Further opportunities do, however, exist to build upon this work and explore if home-working well-being and measures such as attrition and sickness absence are linked in any way and if there are any contrasts between different roles and demographics.

### Limitations

These findings reflect the practice of only one UK ambulance service, which may limit their generalisability. While the findings can be applied to post-pandemic operating models, it is important to note that this was a snapshot of the lived experiences of RCDM staff during the pandemic, and that further research may be needed to determine if the findings remain as they are. Both authors were from ambulance and RCDM backgrounds, albeit the interviewer was not in any managerial or supervision position to create a power imbalance, nor was the interviewer’s primary employment within the operational areas of practice at the time. However, conducting research within communities of which one is already a member can introduce a range of ethical issues. Despite these limitations, the access granted to the sample and the opportunity to gain insight into an unresearched group was positive.

## Conclusion

Like many international services, UK ambulance services use paramedics and nurses to undertake telephone and video assessments of patients calling the 999 emergency services line. The COVID-19 pandemic saw them having to adapt and change their working practices, including moving staff from EOC environments to working from home. This study found no evidence of worse staff well-being due to the move to home working, and in some cases, well-being improved, creating a sense of safety and family security. At the time of the study, it was too early to fully understand the financial implications of home working. However, those staff with long journey times felt more financially secure, even when having to increase home fuel bills, a position that may have changed since the cost-of-living crisis. While professional working relationships appeared to have altered from the more traditional face-to-face, peer-support models, new ways of building relationships with different staff members further afield led to a wider sense of community and teamwork. Anxieties around returning to EOCs were prevalent, but participants noted a sense of inevitability that a blended style of working was required. The findings of this study, although presented in the context of a pandemic, have applicability to post-pandemic operating models and may inform the policies and behaviour of organisations, managers and employees in maintaining positive well-being.

## Acknowledgements

The authors thank the participants for giving up their time to contribute to this research.

## Author contributions

EH drafted the manuscript, with revisions and editing undertaken by MB. EH collected the data, with analysis being undertaken by MB and EH (as described in methods). MB devised the study design and conception. All authors reviewed peer-review comments and approved the final draft of the manuscript. EH acts as the guarantor for this article.

## Conflict of interest

None declared.

## Ethics

Not required.

## Funding

None.

## References

[bibr_1] Ammar A. Mueller P. Trabelsi K. et al. (2020). Psychological consequences of COVID-19 home confinement: The ECLB-COVID19 multicenter study. *PloS One*, 15(11), e0240204–e0240204. https://doi.org/10.1371/journal.pone.0240204. 33152030 10.1371/journal.pone.0240204 PMC7643949

[bibr_2] Association of Ambulance Chief Executives (AACE). (2021). *Ambulance services and the pandemic. A review of 2020–2021*. https://aacesite.s3.eu-west-2.amazonaws.com/wp-content/uploads/2024/03/26110431/Ambulance-services-and-the-pandemic-%E2%80%93-AACE-annual-review-2020-21-FINAL.pdf.

[bibr_3] Barnett J. (2023). *UK’s cost of living crisis will swipe over £2k from Brits’ pockets with some groups expected to feel squeeze of recession even more*. City A.M. https://www.cityam.com/cost-of-living-crisis-to-swipe-2100-from-brits-pockets-as-another-lost-decade-looms/.

[bibr_4] Brady M. & Harry E. (2023). What effects did home-working have on 999 clinician practice from one UK ambulance service during the COVID-19 pandemic? *International Journal of Emergency Services*, 12(2), 343–358. https://doi.org/10.1108/IJES-09-2022-0046.

[bibr_5] Braun V. & Clarke V. (2006). Using thematic analysis in psychology. *Qualitative Research in Psychology*, 3(2), 77–101. http://dx.doi.org/10.1191/1478088706qp063oa

[bibr_6] Brehon K. Niemeläinen R. Hall M. et al. (2022). Return-to-work following occupational rehabilitation for long COVID: Descriptive cohort study. *JMIR Rehabilitation and Assistive Technologies*, 9(3), e39883. https://doi.org/10.2196/39883. 10.2196/39883 PMC9484483 36094442

[bibr_7] Carvello M. Zanotti F. Rubbi I. et al. (2019). Peer-support: A coping strategy for nurses working at the emergency ambulance service. *Acta Bio-Medica: Atenei Parmensis*, 90(11S), 29–37. https://doi.org/10.23750/abm.v90i11-S.8923. 31714498 10.23750/abm.v90i11-S.8923 PMC7233625

[bibr_8] Chen Y. Weziak-Bialowolska D. Lee M. T. et al. (2023). Working from home and subsequent work outcomes: Pre-pandemic evidence. *PloS One*, 18(4), e0283788. https://doi.org/10.1371/journal.pone.0283788. 37014892 10.1371/journal.pone.0283788 PMC10072379

[bibr_9] Chu A. M. Y. Chan T. W. C. & So M. K. P. (2022). Learning from work-from-home issues during the COVID-19 pandemic: Balance speaks louder than words. *PloS One*, 17(1), e0261969. https://doi.org/10.1371/journal.pone.0261969. 35025893 10.1371/journal.pone.0261969 PMC8758108

[bibr_10] Coşkun M. G. Öztürk R. İ. Tak A. Y. et al. (2022). Working from home during the COVID-19 pandemic and its effects on diet, sedentary lifestyle, and stress. *Nutrients*, 14(19), 4006. https://doi.org/10.3390/nu14194006. 36235657 10.3390/nu14194006 PMC9572061

[bibr_11] Denscombe M. (2021). *The good research guide: Research methods for small-scale social research* (7th ed.). Open University Press.

[bibr_12] Dhanasekaran V. Sullivan S. Edwards K. M. et al. (2022). Human seasonal influenza under COVID-19 and the potential consequences of influenza lineage elimination. *Nature Communications*, 13(1), 1721. https://doi.org/10.1038/s41467-022-29402-5. 10.1038/s41467-022-29402-5 PMC8971476 35361789

[bibr_13] Diab-Bahman R. & Al-Enzi A. (2020). The impact of COVID-19 pandemic on conventional work settings. *International Journal of Sociology and Social Policy*, 40(9/10), 909–927. https://doi.org/10.1108/IJSSP-07-2020-0262.

[bibr_14] Galanti T. Guidetti G. Mazzei E. et al. (2021). Work from home during the COVID-19 outbreak: The impact on employees’ remote work productivity, engagement, and stress. *Journal of Occupational and Environmental Medicine*, 63(7), e426–e432. https://doi.org/10.1097/JOM.0000000000002236. 33883531 10.1097/JOM.0000000000002236 PMC8247534

[bibr_15] Giovanis E. & Ozdamar O. (2022). Implications of COVID-19: The effect of working from home on financial and mental well-being in the UK. *International Journal of Health Policy and Management*, 11(9), 1635–1641. https://doi.org/10.34172/ijhpm.2021.33. 33949816 10.34172/ijhpm.2021.33 PMC9808217

[bibr_16] Marczewski K. P. Piegza M. Gospodarczyk A. et al. (2021). Emotional problems and sleep disturbances in paramedics in the era of the COVID-19 pandemic. *Wiadomosci lekarskie*, 74(7), 1754–1757.https://doi.org/10.36740/WLek202107133. 34459782

[bibr_17] Miller E. (2021). The prevalence of stress and burnout in UK emergency ambulance service workers and its impact on their mental health and well-being. *British Paramedic Journal*, 5(4), 62–63. https://doi.org/10.29045/14784726.2021.3.5.4.62. 34421379 10.29045/14784726.2021.3.5.4.62 PMC8341057

[bibr_18] MIND. (2019). *Mental health in emergency services – Our 2019 survey results – Ambulance service*. https://www.mind.org.uk/media-a/4847/2019-survey-ambulance-service-summary.pdf.

[bibr_19] Musgrove E. Bil D. Bridson T. et al. (2022). Australian healthcare workers experiences of peer support during COVID-19: Hand-n-Hand peer support. *Australasian Psychiatry: Bulletin of Royal Australian and New Zealand College of Psychiatrists*, 30(6), 722–727. https://doi.org/10.1177/10398562221128214. 36174218 10.1177/10398562221128214 PMC9527122

[bibr_20] New South Wales Government. (2021). *NSW virtual care strategy, 2021–2026*. https://www.nsw.gov.au/sites/default/files/2023-02/nsw-health-virtual-care-strategy-feb-2022.pdf.

[bibr_21] Okuyan C. B. & Begen M. A. (2021). Working from home during the COVID-19 pandemic, its effects on health, and recommendations: The pandemic and beyond. *Perspectives Psychiatric Care*, 58, 173–179. https://doi.org/10.1111/ppc.12847. 10.1111/ppc.12847 PMC8242705 34003489

[bibr_22] Piotrowski A. Makarowski R. Predoiu R. et al. (2021). Resilience and subjectively experienced stress among paramedics prior to and during the COVID-19 pandemic. *Frontiers in Psychology*, 12, 664540. https://doi.org/10.3389/fpsyg.2021.664540. 34335376 10.3389/fpsyg.2021.664540 PMC8319398

[bibr_23] Platts K. Breckon J. & Marshall E. (2022). Enforced home-working under lockdown and its impact on employee wellbeing: A cross-sectional study. *BMC Public Health*, 22(1), 199. https://doi.org/10.1186/s12889-022-12630-1. 35093054 10.1186/s12889-022-12630-1 PMC8800406

[bibr_24] Shin Y. Hur W. -M. & Park K. (2021). The power of family support: The long-term effect of pre-COVID-19 family support on mid-COVID-19 work outcomes. *International Journal of Environmental Research and Public Health*, 18(19), 10524. https://doi.org/10.3390/ijerph181910524. 34639822 10.3390/ijerph181910524 PMC8508185

[bibr_25] Sommerlad A. Marston L. Huntley J. et al. (2021). Social relationships and depression during the COVID-19 lockdown: Longitudinal analysis of the COVID-19 social study. *Psychological Medicine*, 52(15), 3381–3390. https://doi.org/10.1017/S0033291721000039. 10.1017/S0033291721000039 PMC7844174 33436126

[bibr_26] Tang B. Bragazzi N. L. Li Q. et al. (2020). An updated estimation of the risk of transmission of the novel coronavirus (2019 n-Cov). *Infectious Disease Modelling*, 5, 248–255. https://doi.org/10.1016/j.idm.2020.02.001. 32099934 10.1016/j.idm.2020.02.001 PMC7029158

[bibr_27] Toniolo-Barrios M. & Pitt L. (2021). Mindfulness and the challenges of working from home in times of crisis. *Business Horizon*, 64, 189–197. https://doi.org/10.1016/j.bushor.2020.09.004. 10.1016/j.bushor.2020.09.004 PMC7535863 33041346

[bibr_28] Urch C. E. & George A. J. T. (2020). Let’s stop talking about covid-safe and covid-secure – it’s covid-mitigated. *British Medical Journal*, 370, m3616. https://doi.org/10.1136/bmj.m36.16. 32948602 10.1136/bmj.m3616

[bibr_29] White S. (2022). Refusing to negotiate NHS pay is a false economy. *British Medical Journal*, 379, o3056–o3056. https://doi.org/10.1136/bmj.o3056. 36564080 10.1136/bmj.o3056

